# Physiological network approach to prognosis in cirrhosis: A shifting paradigm

**DOI:** 10.14814/phy2.16133

**Published:** 2024-07-03

**Authors:** Tope Oyelade, Kevin P. Moore, Ali R. Mani

**Affiliations:** ^1^ Institute for Liver and Digestive Health, Division of Medicine UCL London UK; ^2^ Network Physiology Laboratory, Division of Medicine UCL London UK

**Keywords:** cirrhosis, decompensation, liver failure, network physiology, prognosis, systems connectivity

## Abstract

Decompensated liver disease is complicated by multi‐organ failure and poor prognosis. The prognosis of patients with liver failure often dictates clinical management. Current prognostic models have focused on biomarkers considered as individual isolated units. Network physiology assesses the interactions among multiple physiological systems in health and disease irrespective of anatomical connectivity and defines the influence or dependence of one organ system on another. Indeed, recent applications of network mapping methods to patient data have shown improved prediction of response to therapy or prognosis in cirrhosis. Initially, different physical markers have been used to assess physiological coupling in cirrhosis including heart rate variability, heart rate turbulence, and skin temperature variability measures. Further, the parenclitic network analysis was recently applied showing that organ systems connectivity is impaired in patients with decompensated cirrhosis and can predict mortality in cirrhosis independent of current prognostic models while also providing valuable insights into the associated pathological pathways. Moreover, network mapping also predicts response to intravenous albumin in patients hospitalized with decompensated cirrhosis. Thus, this review highlights the importance of evaluating decompensated cirrhosis through the network physiologic prism. It emphasizes the limitations of current prognostic models and the values of network physiologic techniques in cirrhosis.

## 1 INTRODUCTION

Physiological systems comprise multiple connected subsystems interacting to maintain homeostasis in an ever‐changing environment. This interaction may be conveyed via direct anatomical connection or in most cases purely functional or physiological. Thus, disruption in the complexity or connectivity changes the unique and collective inherent ability of the system to adapt and reduces the complexity of various physiological variable (Gallagher & Appenzeller, 1999). This failure to adapt or respond appropriately can be transient and mild, or it can be devastating, as with sepsis and multiple organ failure (Buchman, 2006; Ivanov, 2021). Reduced complexity in physiological variables such as cardiac rhythm (Satti et al., 2019), blood oxygen saturation (Al Rajeh et al., 2021; Gheorghita et al., 2022), and skin or core body temperature (Bottaro et al., 2020; Mowery et al., 2011) is associated with increased mortality. Further, reduced functional connectivity between organ systems significantly and independently predicts survival in cirrhosis (Oyelade et al., 2023; Zhang et al., 2022) or sepsis (Asada et al., 2016, 2019). Understanding the interaction between these units (and/or subunits) may help us to understand the dynamics of complex diseases and direct early intervention to improve outcomes.

Simple models that assess organ systems in isolation remain the typical methods to estimate prognosis in many complex diseases such as decompensated cirrhosis (e.g., MELD, model for end‐stage liver disease), sepsis (e.g., SOFA, sequential organ failure assessment score), and others. These models do not consider the complex interaction between the individual units. Thus, many prognostic models fail to optimize their value.

The network method provides an alternative approach based on the complex interactions between individual organ systems within a physiological system, irrespective of anatomical connections. Network physiology identifies the dynamics of connections between seemingly individual organ systems and improves clinical evaluation and assessment of prognosis (Bartsch et al., 2015; Bashan et al., 2012). Patients with decompensated cirrhosis or sepsis are at high risk of developing multi‐organ dysfunction, failure, and death. Recent advances in network physiology have paved the way for the application of network mapping to physiological data with the hope of early intervention and improved outcomes (Asada et al., 2016, 2019; Oyelade et al., 2023; Tan et al., 2020; Zhang et al., 2022).

Decompensated cirrhosis is a late stage of liver cirrhosis characterized by multiple organ dysfunction with patients developing ascites, hepatic encephalopathy, portal hypertension, kidney failure, cardiomyopathy, abnormal pulmonary function, immune dysfunction, or impairment of circadian rhythms (D'Amico et al., 2022; Geng et al., 2022; Liu et al., 2022; Montagnese et al., 2010; Rodríguez‐Roisin & Krowka, 2008). The development of hepatic decompensation marks a pivotal stage in the clinical evolution of cirrhosis and is associated with poor prognosis. The complex interaction between systems may generate unexpected outcomes with directed therapy (D'Amico, 2014; D'Amico et al., 2006; Ginés et al., 1987; Planas et al., 2006). For example, targeting nitric oxide to regulate the hyperdynamic circulation in patients with decompensated cirrhosis was expected to lead to clinical improvement and yet was associated with mental status deterioration with restlessness, confusion, and disorientation (Cárdenas et al., 2007; Kalambokis et al., 2005). Likewise, the use of albumin plus terlipressin, a synthetic vasopressin analogue, to improve kidney function in patients with hepatorenal syndrome was unexpectedly observed to be associated with pulmonary edema in some patients (Wong et al., 2021). In a recent multinational study aimed at targeted replacement of plasma albumin in patients hospitalized with decompensated cirrhosis and hypoalbuminemia, China et al. (2021) found no significant benefit. However, a significant increase in pulmonary edema was observed in the treatment arm. This evidence, and others beyond the scope of this review, highlights the failure of isolated targeting of unique organ systems without much consideration for the system‐wide physiological context within which such organ system operates.

In the clinical course of cirrhosis, the compensated stage may last many years as the gradual breakdown in the liver's ability to perform its pivotal roles in maintaining homeostasis is balanced by other organ systems. Untreated cirrhosis may then result in total breakdown in liver function following liver injury due to sepsis or other insults and being complicated by other secondary events such as variceal bleeding, ascites, or hepatic encephalopathy. In some, there may be sufficient recovery of liver function following cessation of alcohol consumption in the alcoholic, treatment of hepatitis C in the patient with chronic viral hepatitis, or treatment of autoimmune liver disease by immunosuppression. However, once a patient passes the point of decompensation, the prognosis is often poor and the only definitive treatment option is limited to liver transplantation (Angeli et al., 2018; Gustot et al., 2015).

Regardless, it is important to have and develop accurate scoring systems that predict survival. This is for the prioritization of liver transplantation and enhanced survival of the recipients. Until recently (2021), the MELD‐Sodium (MELD‐Na) score was the gold standard for prognosis in patients with decompensated cirrhosis. The score comprises a calculation based on serum sodium, bilirubin, and creatinine, together with the prothrombin time (international normalized ratio, INR) (Ruf et al., 2005). Currently, the MELD 3.0 has been proposed as a replacement for the MELD‐Na (similar to UKELD) and includes gender, serum albumin, and statistical interaction between albumin, creatinine, bilirubin, and serum sodium with superior prognostic discrimination in cirrhosis (Kim et al., 2021). Advances in our understanding of decompensated liver disease implicate other factors such as age, cholesterol, hospital length of stay, and white blood cell count as determinants of patients' outcome (Kartoun et al., 2017). Thus, as research continues to unravel the physiological depth of cirrhotic decompensation, it is becoming clearer that simply considering a few surrogate biomarkers, especially as isolated independent variables, may not be sufficient for accurate prediction of clinical outcomes. Recent application of the “network approach” in cirrhosis has provided insight and sometimes surprising results beyond the ability of current models, suggesting that the network approach may enhance prognostic modeling in cirrhosis.

In this review, we discuss the central role of liver in the homoeostatic network. Recent advances in the use of network physiology in cirrhosis, especially in terms of prognosis and predicting the response to treatment, are described.

## 2 THE LIVER IS HIGHLY CENTRAL IN THE PHYSIOLOGICAL NETWORK HUB

The liver has direct and indirect synthetic, metabolic, and immune functions. The synthetic function of the liver makes it an essential modulator of microcirculation (through the synthesis of albumin) and hemostasis (through the synthesis of coagulation factors). The liver plays a crucial role in glucose/energy metabolism, and the hepatocytes' oscillatory clock gene expression modulates central circadian rhythms and behaviors (Delbès et al., 2023). The liver is an important systemic barrier and clears a variety of different endogenous (e.g., hormones) and exogenous compounds (e.g., xenobiotics, gut‐derived bacterial lipopolysaccharide endotoxins) with implications in the pathophysiology of diseases. Aside, various recent works have established links between the liver and other organ systems, especially the enteric and nervous systems (the gut–brain axis). Indeed, the translocation of pathogen‐associated molecular patterns (e.g., gut bacterial lipopolysaccharides) into the systemic circulation (due to increased gut permeability or reduced hepatic clearance) remains one of the key drivers of decompensatory events (e.g., encephalopathy) and mortality in patients with liver failure (Arvaniti et al., 2010; Chen et al., 2015; Moreau et al., 2013; Nicoletti et al., 2019; Rainer et al., 2018). The crosstalk between the liver and the nervous system has been shown to regulate the hepatic metabolism of lipid and glucose as well as glycogen storage (Edwards & Silver, 1970; Imai & Katagiri, 2021; Shimazu & Fukuda, 1965). Furthermore, the contribution of gut microbiome dysbiosis to the development, prognosis, and treatment of liver disease has received significant attention in the recent years. Indeed, altered intestinal microbiomes have been linked with the development of various etiologies of liver disease (Bajaj, 2019; Yang et al., 2017). For instance, the development of metabolic associated steatotic liver disease (MASLD) and steatohepatitis was linked with dysregulation in the gut microbiota (Boursier & Diehl, 2015; Zhu et al., 2013) as well as other components of the gut–brain axis (Bhat & Mani, 2023).

While the communication between the gut and the liver is driven mainly by anatomical connections (e.g., directly via the portal vein and the enterohepatic circulation) (Albillos et al., 2020; Wahlström et al., 2016), there are various complex communication pathways between the liver and brain including the neural pathways, humoral signaling, circulating cytokine and monocyte‐to‐brain signaling (D'Mello & Swain, 2011; Stasi, 2021). For instance, the vagal neural pathways, including the afferent and efferent vagal arms, can respectively communicate and trigger pathophysiological changes in the visceral organs to maintain homeostasis (Eftekhari et al., 2014; Ek et al., 1998; Hajiasgharzadeh et al., 2014) or influence brain‐derived motivational states and sickness behaviors (Harrison et al., 2009). Indeed, impaired metabolism and clearance of ammonium ions via disruption to the urea cycle (Wierling, 2014) and reduced enzymatic activities of the glutamine synthase (Tapper et al., 2015) due to reduced liver function or circulatory bypass via portosystemic shunting are hallmarks of decompensated cirrhosis (Deutsch‐Link et al., 2022). The resulting hyperammonaemia combined with systemic accumulation of the gut‐derived false neurotransmitters (e.g., octopamine) (Lam et al., 1973) and endotoxins (Wright et al., 2007) play a role in impaired neural function observed in patients with cirrhosis.

**FIGURE 1:**
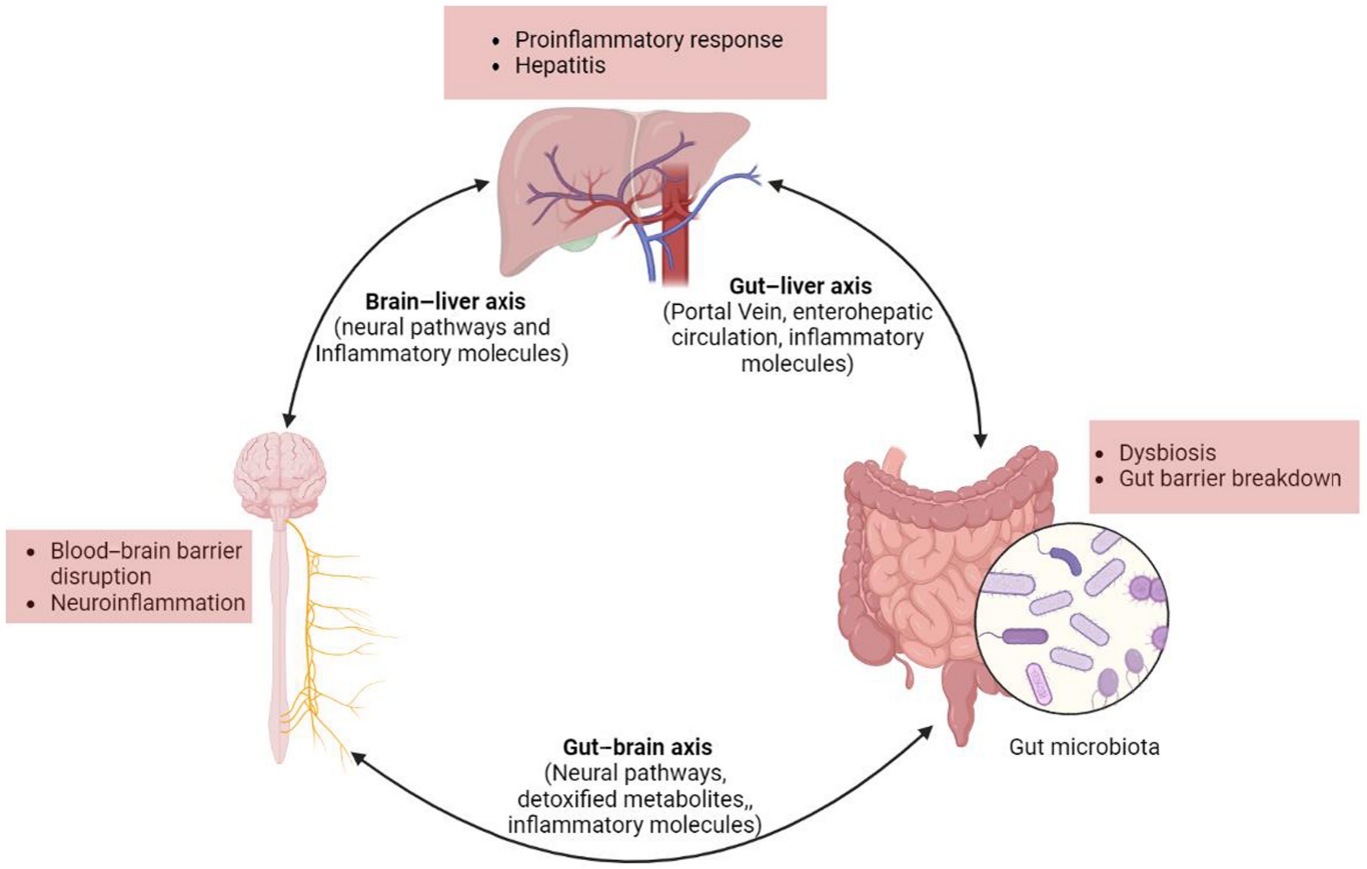
The gut–liver–brain axis is closely connected via diverse pathways and regulates the activities of each other. Image created using Biorender.

Further, reduced clearance of gut‐derived bacterial lipopolysaccharides seen in liver failure leads to a systemic increase in circulating cytokines (IL‐1β, IL‐6, and TNFα) and may induce peripheral as well as cerebral endothelial cells to produce nitric oxide and prostaglandins. The production of these mediators is linked with the activation of sickness behaviors as well as cognitive and behavioral changes (de Paiva et al., 2010; Peng et al., 2012). Circulating cytokines may penetrate via the circumventricular organs of the brain, thereby influencing nervous activities during systemic inflammation linked with liver disease (D'Mello et al., 2009; Miller & Raison, 2016). For instance, in a model of liver disease, circulating monocytes were observed to alter the excitability of neurons and initiate sickness behaviors (D'Mello et al., 2013). Evidence also shows that dysregulation in the gut–brain axis is significantly linked with increased alcohol cravings and decreased social cognition (Carbia et al., 2023) (Figure 1).

These complex interactions between the liver and other organ systems make it an attractive field to be studied within the context of network science in general and network physiology in particular (see Appendix A for more information about the principles of Network Science relevant to physiology). In this review, connectivity between organs is defined as “functional” connectivity, irrespective of anatomically direct links between such systems. Importantly, while these connections between various physiological systems have been inferred (both physiologically and statistically), significant uncertainty persist about the nature of these connections. For instance, it is unclear whether there are intermediate organ systems regulating or regulated by this connectedness or the strength and threshold of connectivity required to maintain a healthy state. These, among other rising questions regarding physiological connections, further underpin the aim of network physiologic methods such as functional and parenclitic network analyses (see Appendix A). This connected role of the liver makes it a good model for network physiology, and continued research along this line is likely to open new frontiers of understanding regarding the critical communication axis or nodes that could be best pharmacologically or nonpharmacologically (e.g., through nerve stimulation, fractal‐like ventilation; Nataj et al., 2018) targeted to improve the prognosis of patients with decompensated cirrhosis.

## 3 CIRRHOTIC DECOMPENSATION—A NETWORK PROBLEM

The ultimate outcome of chronic liver injury is cirrhosis, which is characterized by the replacement of normal liver tissue with scar tissue (fibrosis), leading to the loss of liver architecture and function. With approximately 2 million global annual deaths, chronic liver disease remains one of the major diseases of high epidemiological significance (Cheemerla & Balakrishnan, 2021). This number highlights the complexity of decompensated cirrhosis and the implication of this on clinical management. Indeed, this complexity also makes decompensated cirrhosis a prime candidate for network analysis especially due to its extrahepatic involvements.

The trigger for the decompensation of cirrhosis has been explained by various classical hypotheses including the “vascular underfilling” and “overflow” theories (Lieberman et al., 1970), the “peripheral arterial vasodilation” hypothesis (PAVH) (Schrier et al., 1988), and the “systemic inflammation” hypothesis (SIH) (Bernardi et al., 2015). Irrespective of this, the decompensation event remains a pivotal stage in the clinical history of cirrhosis and is associated with high mortality. While patients with compensated cirrhosis may survive for over 12 years, decompensation results in a significant reduction in survival time to approximately 2 years (D'Amico et al., 2006). Indeed, the risk of mortality has been shown to be significantly linked with the number of organ systems involved in cirrhotic decompensation (D'amico et al., 2014).

Accordingly, decompensation is mainly driven by a clinically significant increase in portal pressure and a decline in liver function (D'Amico et al., 2001, 2006). This combined breakdown often results in jaundice, gastrointestinal bleeding, encephalopathy, and ascites, which are clinical hallmarks of the decompensatory phase of cirrhosis. These events may also herald other extrahepatic complications such as autonomic dysfunction, hepatorenal syndrome, hepatopulmonary syndrome, cirrhotic cardiomyopathy, and rebleeding resulting finally in multi‐organ failure (Dümcke & Møller, 2008; Møller et al., 2010; Oyelade et al., 2020, 2021; Saunders et al., 1981). Curiously, most of the extrahepatic complications of decompensated cirrhosis are not associated with significant structural damage to the involved organ and have been reported to be reversible by liver transplantation (Kim et al., 2004; Liu et al., 2005; Saigal et al., 2013; Wong et al., 2015). These findings further support the need to focus more on organ systems interaction rather than the individual units.

**FIGURE 2:**
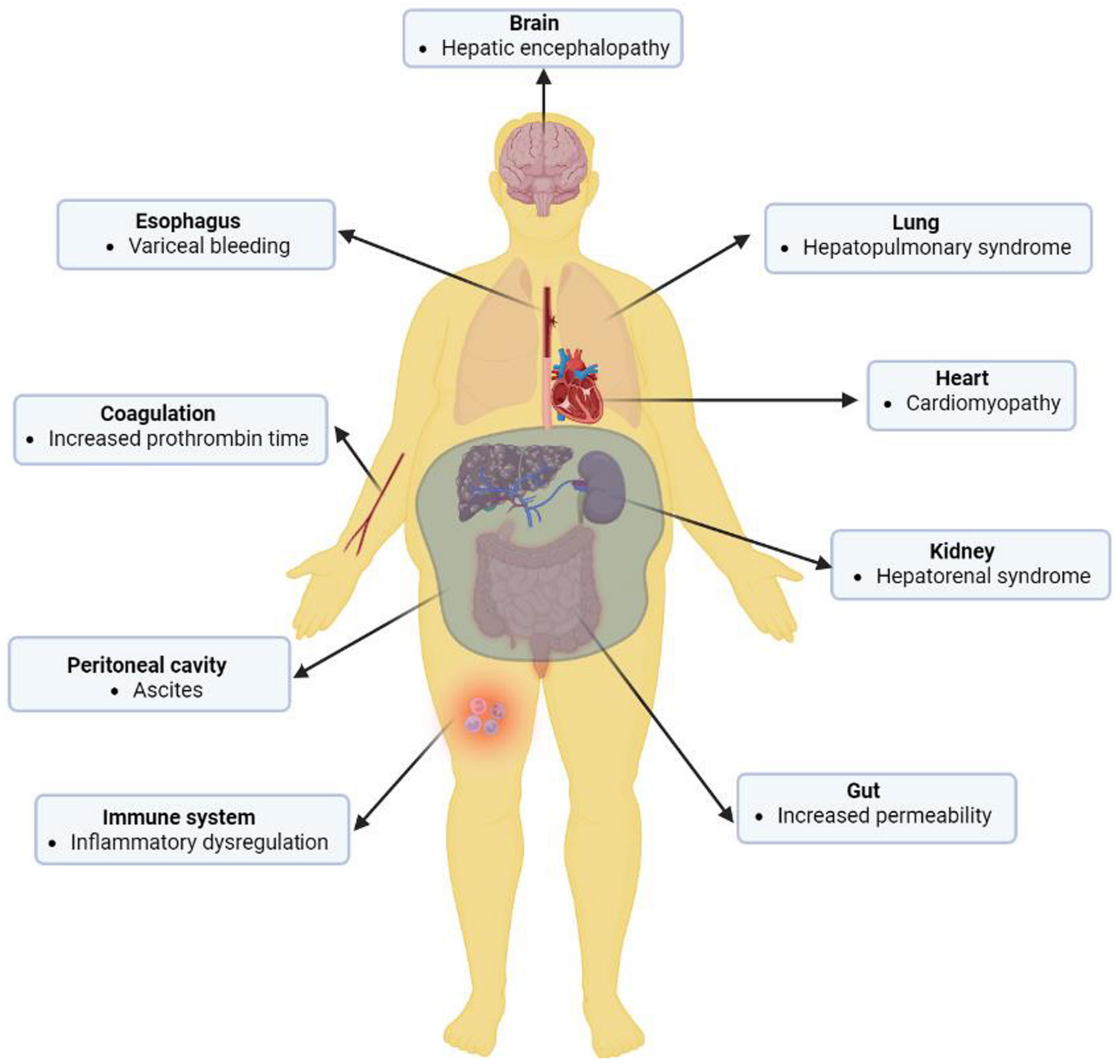
The decompensation stage of cirrhosis is heralded by extrahepatic organ involvement affecting various organ systems including the circulatory system (cirrhotic cardiomyopathy), the nervous system (hepatic encephalopathy and dysregulated autonomic cardiac regulation), the kidney (hepatorenal syndrome), the respiratory system (hepatopulmonary syndrome), digestive system (intestinal injury and increased permeability of the intestinal wall), blood coagulation, and immune system. Also, fluid buildup in the peritoneal cavity (ascites) may result from increased portal tension. Image created using Biorender.

**FIGURE 3:**
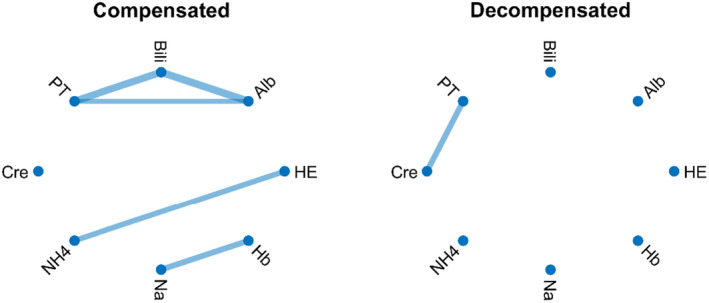
Correlation network map of compensated (left panel) and decompensated (right panel) cirrhosis. Each link shows a statistically significant correlation between the two biomarkers. Alb, serum albumin; Bili, total bilirubin; Cre; serum creatinine; Hb, hemoglobin; HE, hepatic encephalopathy; Na, serum sodium; NH_4_, serum ammonia; PT, prothrombin time. There is more correlation between biomarkers in patients with compensated cirrhosis. Most of the correlation is lost in patients with decompensated cirrhosis. Data were extracted from 106 patients with cirrhosis referred to the University Hospital of Padova (Courtesy of Prof. Sara Montagnese and colleagues) as described in (Zhang et al., 2022). A Bonferroni‐corrected *p*‐value was used to identify statistically significant correlations for network mapping.

Generally, decompensation is a critical stage of cirrhosis characterized by a cascading breakdown of multiple extrahepatic organ systems (Bajaj et al., 2014) (Figure 2). This often culminates in multiple organ failure and a significant increase in mortality risk (Bajaj et al., 2014; Møller & Bendtsen, 2015; Sarin et al., 2014). Indeed, recent studies have shown that decompensated cirrhosis is associated with network disruption as shown in Figure 3. In this figure, statistical correlation analysis between pair of physiological and biochemical biomarkers shows higher correlations between biomarkers in patients with compensated cirrhosis compared with those with decompensated cirrhosis. Specifically, patients with compensated cirrhosis exhibit stronger correlations between albumin, prothrombin time, and bilirubin (representing different aspects of liver function). Intuitively, this correlation is expected in patients who have preserved compensatory mechanisms. The lack of correlation between these components in decompensated patients might indicate uncoupling of physiological mechanisms or distinctive pathophysiology. For example, ammonia levels correlated well with the severity of hepatic encephalopathy (HE in Figure 3) in compensated cirrhosis, which aligns with the classical understanding of hepatic encephalopathy pathophysiology. However, the lack of correlation between ammonia levels and hepatic encephalopathy in decompensated patients indicates that other mechanisms, rather than hyperammonaemia, may play a role in cognitive dysfunction in these patients. These mechanisms might include neuroinflammation and systemic inflammation (Wright et al., 2007), which are not depicted in this preliminary network analysis. Additionally, a connection between creatinine levels and the degree of hepatorenal syndrome in decompensated cirrhosis reflects the fact that in compensated cirrhosis, mechanisms effectively regulate vasodilation and blood pressure, maintaining normal ranges. However, in decompensated cirrhosis, these mechanisms may fail, leading to renal flow impairment and general deterioration (Ojeda‐Yuren et al., 2021). The reason for the correlation between sodium level and hemoglobin level in compensated patients with cirrhosis is not well understood. It might suggest that dilutional anemia may play a role in the pathophysiology of anemia in these patients. However, this needs to be investigated in future studies. Overall, network mapping may provide insight into the pathophysiology of disease and pave the way for future investigations. Importantly, the loss of coordination between organ systems was found to predict the survival of patients with decompensated cirrhosis independent of MELD (Tan et al., 2020; Zhang et al., 2022).

The exact mechanism for physiological network disruption in decompensated cirrhosis is not well understood. However, evidence from experimental (in vitro, ex vivo, and in vivo) studies indicate impaired end organ responsiveness to physiologic signals in animal models of decompensated cirrhosis (Castro et al., 1993; Haddadian et al., 2013; Liu et al., 2022; Ostadhadi et al., 2015). For example, cardiomyocytes, cardiac pacemaker cells, and vascular smooth muscle cells exhibit a blunted response to adrenergic and cholinergic stimuli in experimentally decompensated cirrhosis (Jaue et al., 1997; Liu et al., 2000; Mani et al., 2009; Ostadhadi et al., 2015). Other reports support disruption of the physiological control in decompensated cirrhosis by demonstrating reduced controllability of the physiological subsystems in decompensated cirrhosis using advanced statistical techniques used in the analysis of complex systems (Shirazi et al., 2013; Taghipour et al., 2016). For example, Shirazi et al. (2013) used a computational approach and showed that cardiac rhythm in patients with decompensated cirrhosis keeps a significantly longer memory about past decelerating events compared to those with compensated cirrhosis and healthy volunteers. Intuitively, it is more difficult to control a system that holds a long memory of its past, and thus, increased memory can make the physiological network less adaptable to both environmental and intrinsic changes (Ebadi et al., 2011). To confirm this finding, Satti et al. (2019) used an alternative method (the extended Poincare plot analysis of physiological signals) and showed a significantly longer autocorrelation and memory in patients with decompensated cirrhosis in comparison with compensated cirrhosis and healthy individuals. These findings are in line with impaired physiological control and possibly network disruption in decompensated cirrhosis. However, further investigations are required to determine the mechanism behind the transition from compensated to decompensated cirrhosis in terms of organ systems disconnection.

## 4 TRENDS IN PROGNOSTIC MODEL IN CIRRHOSIS

The trends in prognostic models for liver disease in the past decades provide a basis for a shift in paradigm toward network physiologic and holistic approaches. Once decompensation occurs, liver transplantation remains the definitive treatment option. However, due to scarcity, patients are prioritized based on the severity of liver disease and prediction of survival using prognostic models. The very first model used was the Child–Turcotte–Pugh (CTP) score, developed in 1964 primarily to predict survival in patients undergoing TIPS (transjugular intrahepatic portosystemic shunt) for variceal hemorrhage (Child & Turcotte, 1964; Pugh et al., 1973). CTP is based on five physiological variables, that is, ascites, hepatic encephalopathy, bilirubin, serum albumin, and prothrombin time (previously nutritional status), which are individually graded and combined mathematically into three stages (Child A, B, and C) according to the magnitude of the variables.

The CTP score was dropped because of the subjectivity in the classification of some of the included markers such as ascites and hepatic encephalopathy as well as a limitation regarding the “ceiling effect” since patients could not be classed above Child C even if they have relatively worse severity and prognosis (Tsoris & Marlar, 2019). Also, the CTP score does not include renal function, a crucial aspect of cirrhotic decompensation (Cholongitas & Burroughs, 2012). To overcome these limitations, the MELD (Model for End‐stage Liver Disease) score was proposed as the reference tool for prognostication in patients with liver disease and includes patients' INR (international normalized ratio), total bilirubin, and serum creatinine levels (Malinchoc et al., 2000). However, limitations regarding interlaboratory variability in the measurement of creatinine level and INR as well as gender bias resulted in the proposal of modified MELD scores (Cholongitas et al., 2010). Importantly, modifications of the MELD score have been in the form of the inclusion of more biomarkers shown to have independent prognostic values in the patient population. For instance, the United Kingdom Model for End‐Stage Liver Disease (UKELD) score incorporates serum sodium into the MELD score with improved predictive accuracy (Neuberger et al., 2008). Further, Montagnese et al. (2015) showed a significant improvement in the prognosis value of MELD following the addition of the mean dominant frequency of the patient's electroencephalogram (EEG) and proposed the MELD‐EEG model as a better alternative to MELD alone for predicting survival in patients with cirrhosis. MELD‐Plus score was proposed as a better prognostic model and adds albumin, sodium, white cell count (WCC), total cholesterol, age, and length of hospital stay to the traditional MELD variables (Kartoun et al., 2017). However, the MELD‐plus fails to resolve the gender bias associated with the UKELD and does not incorporate all possible extrahepatic decompensation events observed in critically ill patients with cirrhosis who develop acute on chronic liver failure (ACLF). Thus, Kim et al. introduced the MELD 3.0 in 2021 as the gold standard for short‐term prognosis in decompensated cirrhosis. Specifically, the MELD 3.0 adds gender and serum albumin levels to the MELD‐Na (also UKELD) as well as statistical interactions between albumin‐creatinine and bilirubin‐sodium with a reported increase (~9%) in net reclassification and better discrimination especially reducing the gender disparity associated with MELD‐Na (Kim et al., 2021). Further, for patients with ACLF, these models fail to account for the accompanying increase in the risk of mortality. ACLF is a syndrome associated with a significantly poor short‐term prognosis and is clinically characterized by multiple extrahepatic organ failures in patients with acute decompensation of cirrhosis (Moreau et al., 2013). Consequently, the European Foundation for the Study of chronic liver failure (CLIF) developed the CLIF organ failure (CLIF‐OF) score (Jalan et al., 2014), a derivative of the sequential organ failure assessment (SOFA) score (Ferreira et al., 2001) to capture the poorer prognosis due to the sequential breakdown in organ systems function characteristic of late stage decompensated cirrhosis (Engelmann et al., 2018). Recently, the CLIF‐C MET prognostic model was also developed and positively validated by the CLIF group incorporating biomarkers from metabolomics analysis associated with systemic inflammation, mitochondrial dysfunction, and sympathetic nervous activation (Weiss et al., 2023).

The trends in prognostic models for patients with decompensated cirrhosis have followed the continued inclusion of more organ systems (through representative markers) and proportionately reflect the increasing acceptance of the multi‐organ, extrahepatic implications of decompensated cirrhosis. However, current prognostic scores/models still consider the organ systems as separate, independent units instead of coordinated functioning parts constantly communicating as a network to maintain homeostasis.

## 5 OMICS AND SYSTEM BIOLOGY IN LIVER CIRRHOSIS

As more research continues to clarify the complex pathophysiology of decompensated cirrhosis, attention is being gradually diverted toward a holistic view of prognosis. In recent years, scientists have successfully employed machine learning and artificial intelligence approaches for prognosis in liver disease (Briceño et al., 2014; Garcia et al., 2019; Nishida & Kudo, 2023; Nitski et al., 2021). Various omics analyses have been performed in cirrhosis resulting in new insights into the pathophysiology of cirrhosis as well as biomarkers of significant prognostic values. For instance, blood metabolomics of patients with decompensated and compensated cirrhosis revealed that mitochondrial dysfunction via systemic inflammation may drive organ failure in chronic liver disease (Moreau et al., 2020). Also, Clària et al. (2021) performed an untargeted lipidomic analysis of serum from patients with acute decompensation of cirrhosis with and without ACLF and reported novel lipid fingerprints associated with liver dysfunction and infection. In a metabolomic analysis of urine and serum samples from 211 participants, Bajaj et al. (2019) reported alteration in the bioenergetics, lipid, and protein metabolism in outpatients with cirrhosis. The use of proteomics in alcohol‐related liver disease and viral hepatitis has also shown promising pathogenetic insights as well as prognostic values in various other studies (Niu et al., 2019, 2022; Safaei et al., 2020). Put together, as the pathophysiology of decompensated cirrhosis unravels, the extrahepatic involvement of the disease is beginning to become more appreciated. As this happens, it is imperative that researchers and clinicians need a broader more holistic outlook that could help make sense of the complex shift in physiological dynamics and coupling that possibly drives prognosis and treatment response.

## 6 PHYSIOLOGICAL SIGNALS VARIABILITY IN LIVER DISEASES

Classical physiological interpretation relies on the Cannon principle that human physiology is maintained in a “fairly constant or steady state” (Cannon, 1929). This principle, however, has been debunked due to the continuing discovery that organ systems vary and interact in complex and nonlinear ways across time to achieve and maintain homeostasis in direct response to an ever‐changing environment (stressors) (Gallagher & Appenzeller, 1999; Seely & Macklem, 2004). Therefore, higher variability and complexity of the time series of physiological variables have been interpreted as an outcome of increased organ systems engagement (coupling) with the appearance of regularity linked with the isolation of a system and reduction in adaptability and survival (Pincus, 1994). Thus, various measures, including short‐ and long‐term indexes of heart rate variability (HRV), represent the complex interplay between various spatiotemporal signals from nervous, respiratory, and circulatory systems as well as the regulators of circadian oscillation of core body temperature among others (Shaffer et al., 2014).

Heart rate variability can be computed from electrocardiogram and measures the variation in the time intervals between consecutive heartbeats (R–R interval duration, RRI) (Electrophysiology, Task Force of the European Society of Cardiology the North American Society of Pacing, 1996; Hon, 1965). Generally, higher HRV is linked with health and is interpreted as a higher influences of various organ systems on the heart rhythm. For instance, the short‐term variation of heart rate indexed by the high frequency distribution of power in an ECG recording may be attributed to the coupling of respiration (vagally controlled respiratory sinus arrhythmia) via the autonomic nervous systems with the cardiac cycle (Karemaker, 2009; Schwartz & Andrasik, 2017). Further, the long‐term (24‐h) influence on HRV is linked with the cardiac coupling with the baroreflex loop, renin‐angiotensin pathway, core body temperature, and circadian rhythms (Shaffer et al., 2014). Indeed, recent works have shown a reduction in HRV indices in patients with cirrhosis, which is significantly linked with survival (Miceli et al., 2023; Oyelade et al., 2021). Interestingly, long‐term nonlinear indices of HRV (i.e., standard deviation parallel to Poincare's line of identity, SD2), which is strongly influenced by baroreflex loop and thermoregulation, significantly predicted survival in a study by Bhogal et al. (2019).

**FIGURE 4:**
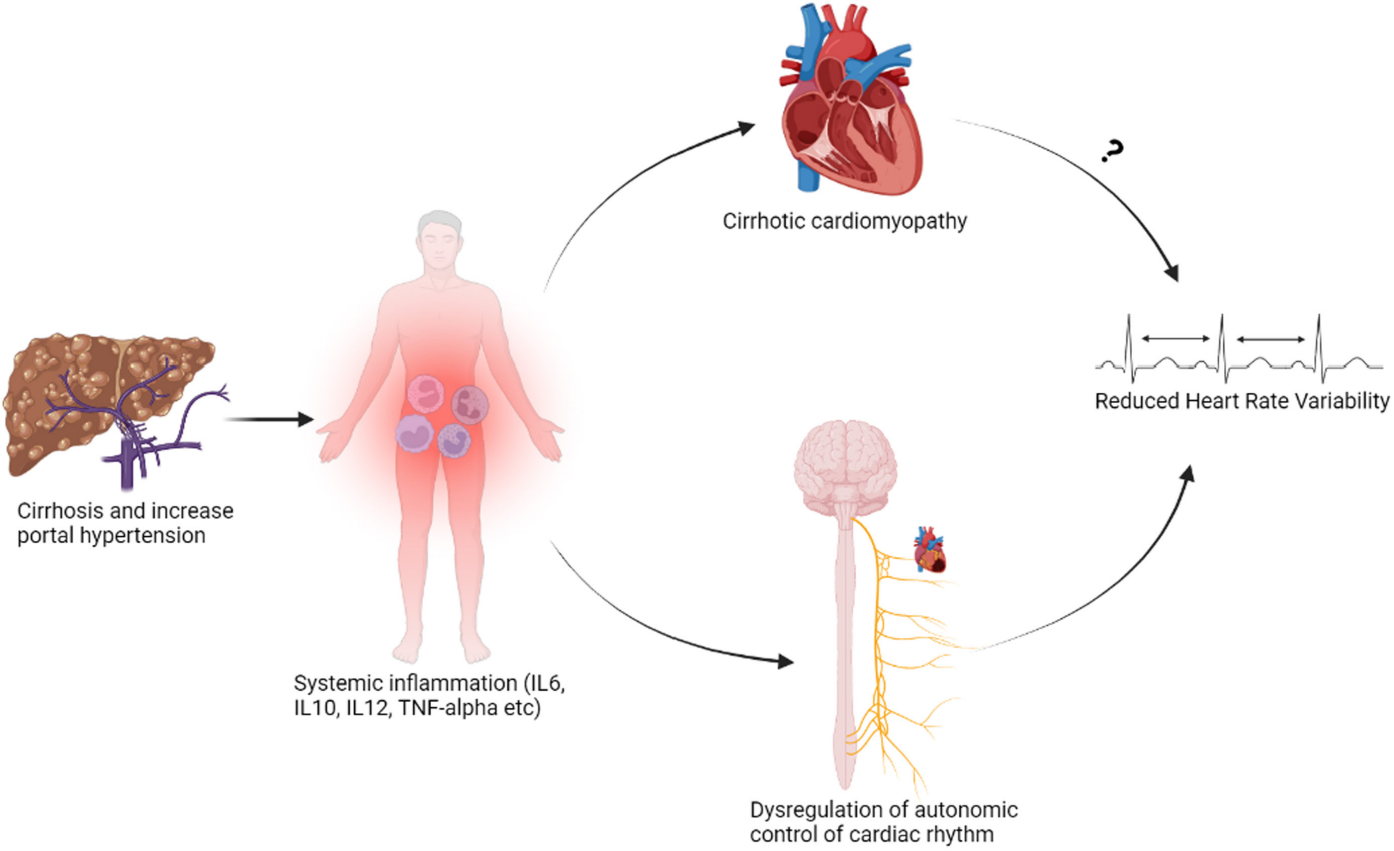
Pathogenesis of reduced heart rate variability (HRV) in cirrhosis is linked with systemic inflammation resulting in disruption to the autonomic (vagal) nervous control of cardiac rhythm. Image created using Biorender.

Mechanistically, the HRV reduction observed in cirrhosis has been linked with systemic inflammation. Although decompensated cirrhosis is linked with cirrhotic cardiomyopathy, it is unclear whether this is associated with HRV change (Abid & Mani, 2022). This is based on the observation that pharmacological interventions (e.g., NO synthase inhibitors or n‐acetylcysteine) restoring cirrhotic cardiomyopathy do not correct reduced HRV in rats with cirrhosis (Abid & Mani, 2022). Alternatively, HRV reduction in cirrhosis has been strongly linked with hepatic encephalopathy and was reported by Mani et al. to correlate with systemic levels of inflammatory biomarkers such as interleukins 6 (IL‐6). Thus, inflammation remains the main driver of HRV reduction in cirrhosis possibly as a consequence of the associated organ system network disconnection (Abid & Mani, 2022; Mani et al., 2009; Williams et al., 2019) (Figure 4).

While HRV provides a relatively simple, noninvasive measure of cardiac connectivity to other organ systems, it is limited by the availability of clean ECG data with a high signal‐to‐noise ratio. This may be impossible in cirrhosis patients with abnormal heart cycles (e.g., due to premature ventricular beats) or those that are active. In such patients, heart rate turbulence (HRT), which indexes the autonomic and baroreflex regulation of heart rhythm following a premature ventricular contraction (Bauer et al., 2008; Schmidt et al., 1999), is a viable alternative. Indeed, the prognostic value of heart rate turbulence was recently investigated. Specifically, the turbulence onset was found to predict 12‐month survival in patients with cirrhosis independent of MELD and CTP scores (Oyelade et al., 2020).

In addition, variation in the body (skin or core) temperature is a complex process regulated by the nervous system (the hypothalamic thermoregulatory center based on stimuli from thermoreceptors) in response mainly to the circadian rhythm and the environment. Body temperature variability is driven by the interplay between hormonal, autonomic, vascular, and metabolic systems as well as systemic inflammatory response (Karthikeyan et al., 2012; Tansey & Johnson, 2015). Thus, body temperature variability may reflect the influence of disease state on the connectivity of the various systems involved in the thermoregulatory pathway (Mani et al., 2018). Indeed, body temperature analysis has been assessed in both patients with cirrhosis and animal models (Garrido et al., 2017; Mani et al., 2018; Satti et al., 2019). Similar to HRV analysis, the entropy of temperature signal fluctuations correlates with the amount of information in temperature signals according to the basic concept of information theory (Pincus, 1991). The physiological basis for information processing within the context of thermoregulation involves maintaining a dynamic balance between heat loss (e.g., due to vasodilatation) and heat gain (e.g., metabolism, thermogenesis), as well as circadian changes in core body temperature. Previous studies have shown that entropy analysis of body temperature fluctuations can distinguish the severity of diseases. For example, core temperature fluctuation analysis in cirrhotic rats indicated that acute endotoxin injection significantly reduced core body temperature entropy and was associated with mortality (Mani et al., 2018). According to Pincus, reduced entropy of a physiological signal may indicate partial uncoupling of the regulatory control network (Pincus, 1994). Thus, reduction on core body temperature fluctuation entropy (i.e., increased temperature signal regularity) can be interpreted as reduced functional connectivity of thermoregulatory mechanisms. Studies on humans have shown similar results. Reduced body temperature entropy in critically ill patients is associated with poor survival (Drewry et al., 2013; Papaioannou et al., 2012). Likewise, in patients with cirrhosis, reduced short‐term variability but not the absolute values of skin temperature in patients admitted with cirrhosis predicted 12‐month survival independent of MELD and CTP scores (Bhogal et al., 2019; Bottaro et al., 2020). To fully elucidate the physiological interpretation of temperature variability, dynamic network mapping (e.g., using transfer entropy analysis) can be carried out in the future.

Put together, time series of physiological variables such as heart rate and skin temperature vary nonlinearly in response to various cues from various sources (via feedback loops) and remain reliable indices of the complex interplay between various organ systems coupling (or lack of).

## 7 NETWORK PHYSIOLOGY FOR PROGNOSIS IN LIVER CIRRHOSIS

**FIGURE 5:**
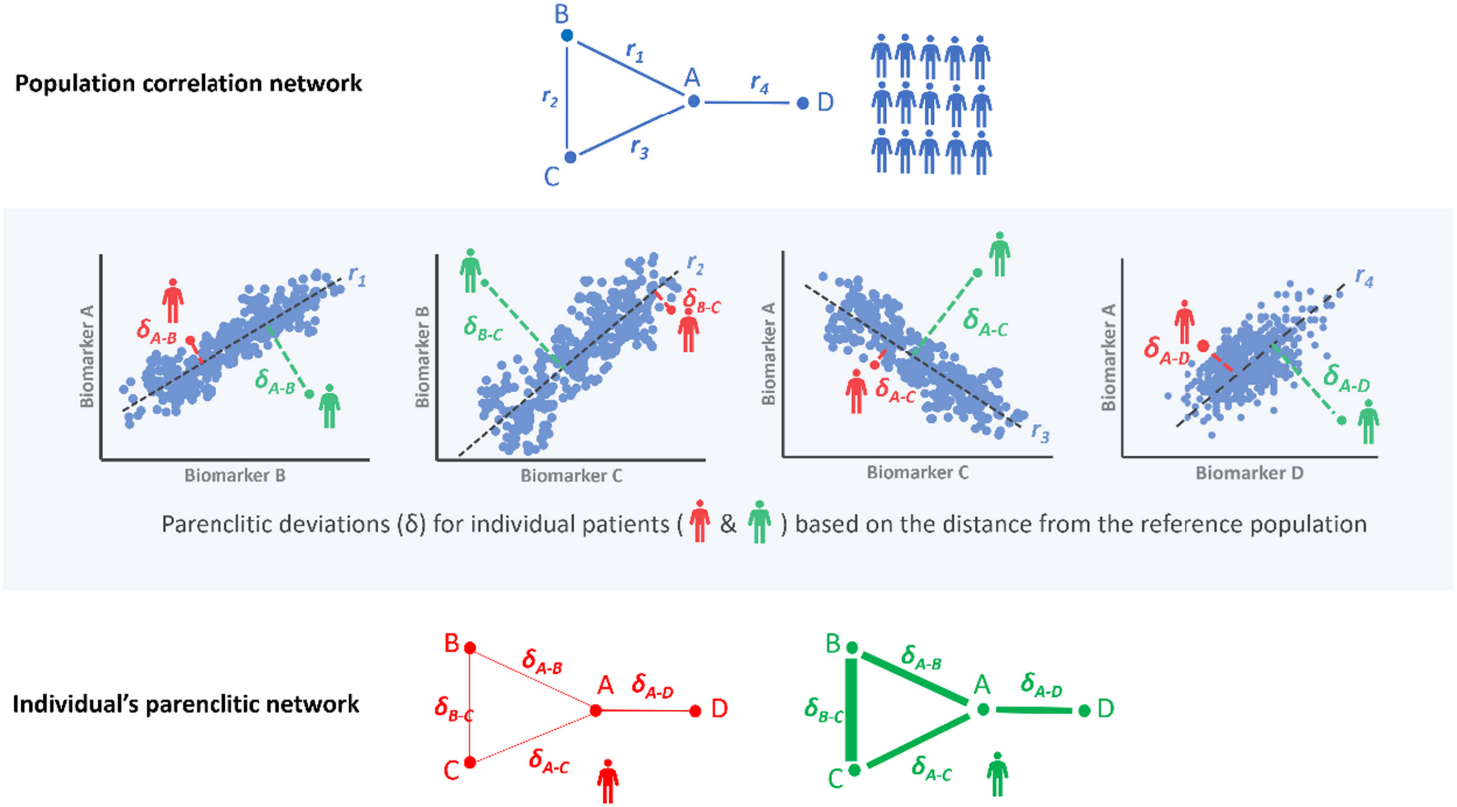
A schematic representation of the Parenclitic network mapping method. Top panel: The network mapping of the reference population (i.e., survivors) utilized correlations between biomarker pairs (e.g., A–B, A–C, B–C, and A–D). Individual reference data points are depicted as blue dots, while black regression lines represent the expected relationship models. Notably, r1, r2, r3 and r4 signify statistically significant correlation coefficients. Middle panel: For individual patient network mapping, a parenclitic approach was adopted. This method assesses how individual patients deviate from the anticipated relationships between variables within the reference population. Essentially, parenclitic deviation indicates the extent to which an individual's biomarker levels differ from the expected model. In this instance, the patient depicted in red exhibits closer alignment with the reference population compared with the green patient in terms of biomarker correlations. Consequently, the green patient displays a higher parenclitic deviation (δ) than the red patient. Lower panel: The parenclitic network map showcases nodes A, B, C, and D, with edges weighted (represented by thickness) based on the extent of deviations from the models for two individual patients (red and green). Thicker edges for the green patient indicate greater parenclitic deviation, reflecting reduced functional connectivity between biomarkers. Conversely, the red patient displays lesser parenclitic deviation, indicating a closer alignment with the reference model and heightened functional connectivity between biomarkers.

The network analysis approaches have recently been successfully applied to cirrhosis patients. For instance, Tan et al. (2020) used correlation network mapping of organ system connectivity in cirrhosis to show that lack of correlation between various biomarkers independently predicts 3, 6, and 12 months of survival. However, the model employed was built on network mapping at the population level, limiting the interpretation of the findings for individual patients. To extend this observation further, Zhang et al. (2022) mapped the physiological network of individual patients with cirrhosis using parenclitic network analysis. The parenclitic network technique was first introduced in 2014 by Zanin et al. (2014a) for assessment of gene expression analysis. This method measures the deviations of an individual patient's organ systems network map from expectations modeled on a reference population (e.g., survivors, responders, healthy volunteers). For instance, pairwise correlations between various biomarkers (A, B, C, and D in Figure 5) from a reference population (e.g., healthy, matched participants, survivors, treatment responders, or patients that showed specific responses to treatment, etc.) are computed to establish a reference regression line. The significantly correlated pairs based on Bonferroni‐corrected *p*‐values can then be combined into a network of correlated variables. The orthogonal deviations of the individual patient's pair of biomarkers from the reference regression lines are then computed and used to weigh their overall parenclitic network map (Figure 5). Thus, the individual patient's parenclitic network map provides the measure of how “deviated” from the physiological norm the patient is and may provide information regarding the health state (disease severity), prognosis, and likelihood to respond to specific treatment regimes.

Zhang et al. (2022) employed parenclitic network analysis of routine clinical variables in patients with cirrhosis and reported that such network mapping can independently predict one‐year survival and provides novel insights into the pathophysiology of cirrhosis not captured by the MELD score. Specifically, patients' physiological deviations along the albumin‐bilirubin and albumin‐prothrombin time significantly predict 12‐month survival and improve the prognostic performance of MELD (Zhang et al., 2022). These findings go along with the idea that higher organ system discoordination is associated with poorer outcomes in patients with cirrhosis. One method for detecting network connectivity involves utilizing network topology indices like network degree distribution (see Appendix A), which assesses coordination among various systems based on the number of edges connected to each node. Alternatively, node importance (centrality) can measure network topology. For example, when centrality indices were applied to predict outcomes in cirrhosis, it was found that those patients with more important nodes in the network had a greater chance of survival (Zhang et al., 2022).

**FIGURE 6:**
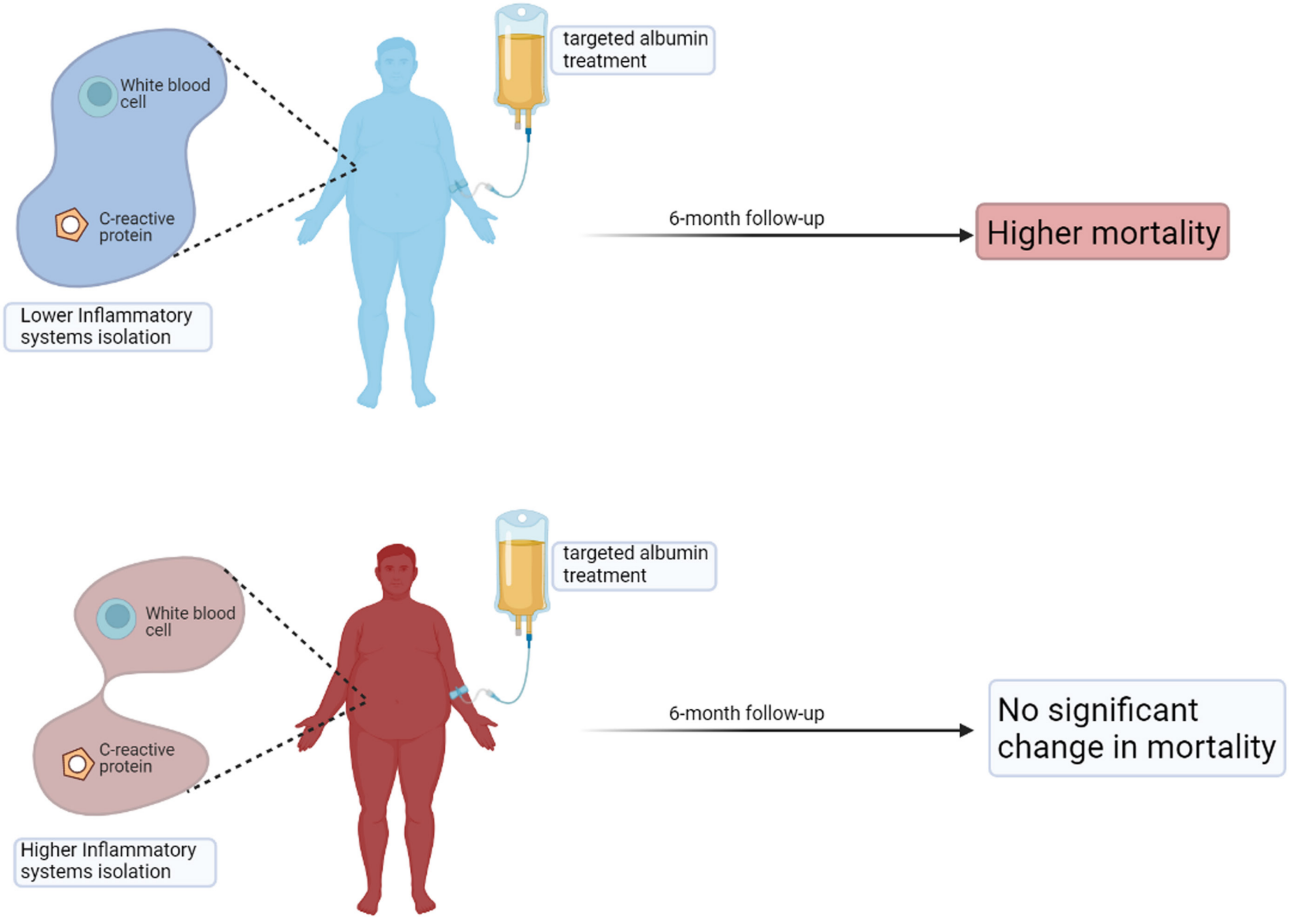
According to the analysis of organ system connectivity using a parenclitic network, patients with lower network disconnection in the inflammatory pathways are more likely to be armed by increased albumin infusion compared with patients with higher inflammatory system isolation for which infused albumin did not result in a significant difference in mortality (see Oyelade et al. 2023, for more). Image created using Biorender.

Further, these results are recently validated by applying the parenclitic network analysis to a larger and more diverse patient population of 777 hospitalized patients across the United Kingdom with decompensated cirrhosis (China et al., 2021). This study further confirms that parenclitic network analysis has prognostic values and augments the predictive power of MELD. It was also found that this method can predict patients unlikely to benefit from increased short‐term targeted albumin treatment. Specifically, the loss of coordination between inflammatory regulators (white blood cell count, WCC, and C‐reactive proteins, CRP) was significantly more pronounced in patients that survived better following targeted albumin infusion. Generally, it is expected that the level of systemic CRP and WCC would be correlated in most patients due to the functional link between the two biomarkers. However, network mapping showed that this association is only intact in survivors and not in the non‐survivors. Indeed, serum albumin level is generally reduced in patients with cirrhosis and has been shown to be an independent predictor of mortality (Bai et al., 2019). Interestingly, various studies have shown that increased albumin does not alter the risk of mortality in cirrhosis (China et al., 2021, 2022). However, network analysis identified that while increased targeted albumin infusion did not have a significant effect on mortality of patients with higher inflammatory system network isolation, patients with stronger coupling between their inflammatory markers (CRP and WCC) were negatively impacted (Oyelade et al., 2023) (Figure 6). This observation is hypothetically a result of a shift in the delicately balanced physiological homeostasis in patients hospitalized with decompensated cirrhosis possibly caused by increased albumin infusion. Thus, the systemic compensatory mechanisms present in patients with better network coupling may have been disrupted by albumin replacement therapy. On the contrary, as this coupling was already lost in the other group, albumin infusion was observed to have no significant effect on overall survival.

The result of this study provides further information on the critical role of inflammatory dysregulation in cirrhotic decompensation and patients' prognosis and corroborates classical works and findings about CRP and WCC. For instance, the role of CRP (as an acute phase protein) in acute inflammatory response is destabilized in cirrhosis with a relatively higher basal CRP level resulting in attenuated response during infection. The implication is that CRP, although a relatively good predictive biomarker of inflammation, is not suitable as such in patients with liver cirrhosis (Pieri et al., 2014). Indeed, in a recent study of 140 patients with hepatitis B‐related decompensated cirrhosis, Zhu et al. (2017) show that CRP predicted 1‐month survival independent of MELD. However, in another study of mixed‐etiology decompensated cirrhosis, while CRP was reported to be significantly linked with infection, only the neutrophil‐to‐lymphocyte ratio predicted survival in patients hospitalized for cirrhotic decompensation (Kwon et al., 2015). In sum, these findings can help drive personalized treatment (e.g., targeted albumin therapy) based on individual patient's network connectivity. Importantly, network mapping is very accessible as it can be constructed from routine clinical and laboratory data, which is available in most healthcare settings globally. Potentially, the mapping software could also be developed into a simple mobile application for bedside clinical use in the future. The application of network mapping in personalized therapy can be explored for other interventions, such as responders versus nonresponders to beta‐blocker therapy for portal hypertension.

## 8 NETWORK PHYSIOLOGY IN OTHER DISEASES

Sepsis is often an important cause of deterioration and mortality in patients with cirrhosis. Assessment of organ systems connectedness has been applied in patients with sepsis as well as critically ill patients admitted to the ICU. For instance, to validate a previous study that showed a similar result (Grogan et al., 2004), Norris et al. assessed HRV and cardiac isolation in over 2000 patients admitted to the ICU and reported that reduced HRV and cardiac uncoupling is a strong predictor of all‐cause mortality. Indeed, cardiac isolation was also reported to be linked with systemic inflammation and multiple organ failure in these patients (Norris et al., 2006). Various HRV indices were used in patients admitted to the ICU specifically for sepsis and have been reported to predict septic shock (Ahmad et al., 2009; Chen & Kuo, 2007; de Castilho et al., 2017) as well as mortality (Bodenes et al., 2022; Chen et al., 2008; Garrard et al., 1993). In a systematic review by de Castilho et al. (2018) HRV was reported to be reduced in sepsis and predictive of mortality.

Further, variability (entropy) in oxygen saturation (SpO_2_) was recently assessed by Gheorghita et al. (2022) in critically ill patients with sepsis showing that SpO_2_ entropy can predict mortality independent of Age, SOFA score, and mean SpO_2_. This work corroborated a previous work by Bhogal and Mani (2017) which showed that variability in oxygen saturation carries information about organ systems uncoupling that possibly drives aging. Other authors have assessed variability in core body and skin surface temperature in patients with sepsis. Indeed, wavelets and multiscale entropy analysis of body surface temperature were reported to discriminate patients with systemic inflammatory response syndrome (SIRS), sepsis, and septic shock (Papaioannou et al., 2012).

Further, Asada et al. (2019) performed a correlation network analysis of clinical variables representing various organ systems to assess their connectivity and show that lack of correlation of the cardiovascular system with hepatic and coagulation systems is linked with significantly poorer survival in critically ill patients admitted to intensive care unit (ICU). In another study involving 570 ICU patients, the stability of organ systems network clusters was analyzed based on principle component analysis and showed a generally high organ system disconnection and systemic instability in critically ill patients that did not survive ICU stay (Asada et al., 2016).

In general, assessment of organ system uncoupling via variability measures of physiological variables or network mapping provides valuable insights regarding the course of complex diseases with multiple organ involvement. Of note, these insights are usually not available using traditional statistical methods or machine learning and artificial intelligence. Network physiology is an emerging field, and novel network mapping methods can expand the depth and magnitude of the interactions we can explore in physiological systems. The number of systems assessed may depend on the methodology applied for network mapping, availability of data, and computational cost. While it is generally believed that assessing more systems provides a better physiological understanding of the interactions, the methodology of network mapping can identify the most significant interactions within the complex physiological network. For example, in parenclitic network mapping (Figure 5), the number of interactions can be reduced by setting a *p*‐value on the correlation between physiological/biochemical parameters (Zhang et al., 2022). Apart from methodology, data availability is also important for assessing physiological network complexity. For example, if the data source is from metabolomic or transcriptomic analysis, rich data can expand network mapping. However, such rich data is not available for most cohorts, and thus, in this review, we only discussed studies on network mapping based on routine physiological signals (e.g., heart rate) and biomarkers (e.g., Albumin). It appears that multiple system interactions can also be expanded to multilayer complexity by connecting functional physio‐markers with omics data. However, this needs to be carried out in future studies.

## 9 LIMITATIONS

Despite its usefulness, some universal limitations of the network approach to physiology have been highlighted. Firstly, quantification of the dimension and dynamics of network physiology relies heavily on the availability of relevant data (Barabási et al., 2011; Cohen et al., 2021). Although some of these data could be generated using the omics approach, the method is relatively expensive and may not be feasible in many clinical settings around the world. Thus, the current approach of using routine clinical data for network analysis is a relatively simple, useful, less expensive, and highly accessible method. For the assessment of organ systems connectivity using HRV, entropy, and other measures of variability of physiological variables, the main limitation remains the heterogeneity of the methods used, which limits meaningful interpretation, generalization, and clinical applicability (Oyelade et al., 2021).

Also, while static network approaches (e.g., correlation and parenclitic), which provide a cross‐section of the interaction at a single time point is feasible with routine data, dynamic network approach, which provides the network change across time based on causal links require relatively more sophisticated datasets not currently standard in most clinical settings. The assessment of dynamic networks is especially important since interacting organ systems generate information at varying time scales (from milliseconds to hours) with associated differences in dynamic outputs (random, stochastic, oscillatory, etc.) with transient information sharing corresponding to internal and external challenges to the overall system (Bartsch & Ivanov, 2014; Ivanov, 2021). This “fleeting,” multiscale coupling may elucidate a crucial juncture in the dynamic network of the system, which is important for proper understanding of the current physiological state as well as predicting future changes (Ivanov, 2021). Indeed, the use of causal indices based on mathematically more sophisticated methods may provide further insights and should be the focus of future research in the field. Most importantly, because the field of network physiology is still developing with application in various diseases (Berner et al., 2022; dos Santos et al., 2022; Hall et al., 2024; Legault et al., 2024; Lehnertz et al., 2023; Rizzo et al., 2023) and scenarios including in sports and sleep analysis among others (Antonacci et al., 2023; Difrancesco et al., 2023; Ganglberger et al., 2023; Mangalam et al., 2024; Marsh et al., 2023; Sides et al., 2023), the methods available for detecting physiological connectivity as well as for quantifying such connectivity will depend on the nature of the research question being assessed, the type, quality, and quantity of data available as well as the technical repertoire at the disposal of researchers.

## 10 CONCLUSION AND PROSPECT: ALL ORGANS, ALL TIME

Despite current limitations associated with data availability and techniques, the clinical usefulness of network physiology in chronic diseases such as decompensated cirrhosis, sepsis, and critical illness is self‐evident and provides new valuable insights to researchers. In fact, because of its ability to detect and quantify physiological connections (or lack of), network physiology has provided quantitative evidence to support previously clinically reported but unexplained pathophysiological observations as shown recently for CRP and WCC (Oyelade et al., 2023). Indeed, the focus of future work would be to further validate and standardize the network analysis based on models built on highly representative reference populations to accelerate translation into clinical practice. Indeed, translation in the form of bedside mobile applications may augment clinical management of patients especially when considered in combination with other established clinical variables or prognostic models. The application of network analysis, especially the parenclitic network, which detects network deviation for individual patients may aid targeted and personalized treatment based on clinical disconnection along unique physiological axes.

Thus, as we march into the brave new world of big data, artificial intelligence, and personalized medicine, finding the pathophysiological needle in the complex haystack of dynamically interacting organ systems in decompensated cirrhosis and other complex diseases might be driven by a deep understanding of the network characteristics of the individual patients based on data from “all organs at all times.”

Irrespective of the current limitations, the future of diagnosis and prognosis in cirrhosis may be “network physiologic” in nature.

## FUNDING INFORMATION

The authors declare that the research was conducted in the absence of any commercial or financial relationships that could be construed as a potential conflict of interest.

## CONFLICT OF INTEREST STATEMENT

None.

## Data Availability

Data are available upon request from the corresponding author.

## APPENDIX A An Introduction to the Principles of Network Science for Application in Physiology

### Introduction

Network physiology is an emerging interdisciplinary scientific field that investigates the topology of functional interactions between various components of physiological systems in health and disease. In network physiology, physiological signals are used in conjunction with computational techniques to gain insights into the network of interactions between different physiological systems. This differs from the traditional reductionist approach in physiology, wherein subsystems are typically studied in isolation. This approach is important as the human body is “an integrated network where complex physiological systems, each with its own regulatory mechanisms, continuously interact, and where failure of one system can trigger a breakdown of the entire network” (Bashan et al., 2012). This approach has the advantage of facilitating the development of methodologies that examine integrated physiological functions holistically for application in healthcare (Jiang et al., 2021). To fully take advantage of this approach, familiarity with the principles of network science is helpful. This appendix provides a summary of the principles of network science relevant to network physiology.

### Principles of network science

Network science is a multidisciplinary field that studies complex networks such as social networks and physiological networks. The following terminologies are essential for in‐depth understating of network science:

#### Nodes and edges

Networks consist of nodes (or vertices) and edges (or links). Nodes represent entities, while edges represent the relationships or interactions between them. The figure below displays nodes (A, B, C, D, and E) in red and edges (a, b, c, d, and e) in blue.

#### Undirected versus directed networks

Undirected networks represent symmetric relationships where connections between nodes have no inherent direction, while directed networks represent asymmetric relationships with edges indicating specific directional connections between nodes.

#### Degree

The degree of a node is the number of edges connected to it. In directed networks, there are in‐degrees and out‐degrees that count incoming and outgoing edges, respectively.

#### Path and distance

A path is a sequence of edges connecting a series of nodes. The distance between two nodes is the length of the shortest path connecting them.

#### Centrality measures

These metrics determine the importance of a node within a network. There are different types of centrality measures, such as degree centrality (i.e., the number of connections a node has) and closeness centrality (i.e., average shortest path length from a node), which are applied depending on the context of the network.

#### Network topology

The arrangement of nodes and edges in a network is called network topology. Different networks may exhibit diverse topologies, such as random networks (where the nodes are connected randomly), hierarchical networks (characterized by its layered arrangement, where nodes at higher levels have overarching influence over nodes at lower levels), and scale‐free networks (where there are a few highly connected hubs and many nodes with few connections).

#### Network classes

Depends on the context that is presented by the network, networks can be categorised into different classes:

##### Physical networks

This type of network represent physical connections between nodes such as transport maps and network of anatomical connections (e.g., anatomical neuronal network, vascular network, etc.).

##### Functional networks

This type of network represents interactions or connections between functional units, such as the network of information transfer among members in social networks. For example, in neuroscience, a functional brain network represents the connections between different brain regions based on patterns of activity or functional connectivity observed in neuroimaging data (e.g., fMRI or EEG) (van Diessen et al., 2015). Nodes correspond to brain regions, and edges represent statistical dependencies or correlations between their activities. Functional networks play a unique role in network physiology and have been used in different physiological and pathological contexts (Jiang et al., 2021).

##### Parenclitic networks

A parenclitic network is a type of network that maps the deviations of individual data points from a reference model (Zanin et al., 2014). The parenclitic network approach allows researchers to focus on abnormal deviations, providing insights that may be missed by traditional network analysis methods. It is particularly useful in identifying subtle but significant deviations that could be indicative of underlying health issues or other conditions. Application of parenclitic networks is discussed in this review (see Figure 3).
